# Weight Change Is Associated with Osteoporosis: A Cross Sectional Study Using the Korean Community Health Survey

**DOI:** 10.3390/ijerph182413368

**Published:** 2021-12-19

**Authors:** Hyo-Geun Choi, Bong-Cheol Kwon, Soo-Hwan Yim, Hyun Youk, Jung-Woo Lee

**Affiliations:** 1 Department of Otorhinolaryngology-Head & Neck Surgery, Hallym University College of Medicine, Anyang 14068, Korea; pupen@naver.com; 2 Hallym Data Science Laboratory, Hallym University College of Medicine, Anyang 14068, Korea; 3 Department of Orthopaedic Surgery, Hallym University College of Medicine, Anyang 14068, Korea; bckwon@hallym.or.kr; 4 Department of Neurology, University of Ulsan College of Medicine, Gangneung 25440, Korea; yimsh3687@gmail.com; 5 Department of Emergency Medicine, Wonju College of Medicine, Yonsei University, Wonju 26426, Korea; yhmentor@gmail.com; 6 Bigdata Platform Business Group, Wonju Yonsei Medical Center, Yonsei University, Wonju 26426, Korea; 7 Department of Orthopaedic Surgery, Wonju College of Medicine, Yonsei University, Wonju 26426, Korea

**Keywords:** body weight changes, weight gain, weight loss, cohort studies, osteoporosis

## Abstract

The purpose of the present study was to analyze the associations between weight change and osteoporosis in Korean adults. Methods: Data from the 2016 Korean Community Health Survey were analyzed. A total of 159,741 participants who were ≥40 years of age were included. The histories of osteoporosis were surveyed in two ways: ‘osteoporosis for entire life’ and ‘current osteoporosis’. The participants were grouped into three categories for simplification as follows: ‘Weight L&M’ (Tried to lose weight or Tried to maintain weight), ‘Weight gain’ (Tried to gain weight), and ‘Never tried’. Additionally, we analyzed their relationship with obesity using the BMI. Results: The adjusted ORs for ‘osteoporosis for entire life’ were 1.20 (95% confidence interval [CI] 1.13–1.27) in the Weight L&M group and 1.83 (95% CI 1.64–2.05) in the Weight gain group. The adjusted ORs for ‘current osteoporosis’ were 1.16 (95% CI 1.08–1.25) in the Weight L&M group and 1.77 (95% CI 1.54–2.02) in the Weight gain group. Conclusions: Compared to the Never tried group, being in either the Weight L&M or Weight gain groups showed a significant impact on the possibility of osteoporosis.

## 1. Introduction

Osteoporosis is one of the most common bone diseases, characterized by a reduction in bone mass and compromised bone strength [1]. In Koreans aged ≥50 years, 22.4% had osteopenia and 47.9% had osteoporosis, and incidence gradually increased with age [2]. Moreover, osteoporosis predisposes a person to an increased risk of fracture [1].

The prevalence of obesity among adults is rapidly increasing, and the size of the population trying to lose weight is also increasing. The share of US adults who tried to lose weight increased from 43% in 2007 to 49% in 2016 [3]. The pooled estimate for the prevalence of weight loss attempts in the global general population was 41.5% in meta-analysis (95% confidence intervals [CI] 38.7 to 44.4%; Q = 27,947, *p* < 0.001) [4]. A higher prevalence of weight loss attempts among adults was observed in overweight and obese persons and women.

Several studies have reported that weight loss can cause a decrease in bone mineral density (BMD) [5,6,7,8]. In the Study of Osteoporosis Fractures, older women with weight loss showed increased rates of hip-bone loss, irrespective of current weight or intention to lose weight [5]. A systematic review found that weight reduction results in reduced BMD at the hip but has less effect on the spine [7]. However, many previous studies failed to distinguish between intentional and unintentional weight loss. Unintentional weight loss is frequently associated with comorbidities and poor health, whereas intentional loss may have bone-sparing effects [9]. There are relatively fewer studies about changes in bone health in people who try to gain weight. Because we were unable to locate a study on changes in BMD due to weight gain intention, we refer here to studies of specific diseases. A systematic review reported that spine BMD was lower in bulimia nervosa subjects compared with healthy controls [10]. There are also no studies on the intention of weight gain and the occurrence of osteoporosis. Thus, the understanding the potential effects of intentional weight change on bone health is incomplete.

We hypothesized that intentional weight loss would result in osteoporosis in adults. Additionally, we thought that the attempt to gain weight would also affect osteoporosis. Therefore, we estimated the associations between weight change and osteoporosis using the Korean Community Health Survey (KCHS).

## 2. Materials and Methods

### 2.1. Study Population and Data Collection

This study was approved by the Institutional Review Board of Korea Centres for Disease Control and Prevention (KCDC) using KCHS (IRB No. 2010-02CON-22-P, 2011-05CON-04-C, 2012-07CON-01-2C, 2013-06EXP-01-3C,2014-08EXP-09-4CA, and 2016-10-01-T-A). A detailed description is presented in our previous studies [11,12].

**Figure 1:**
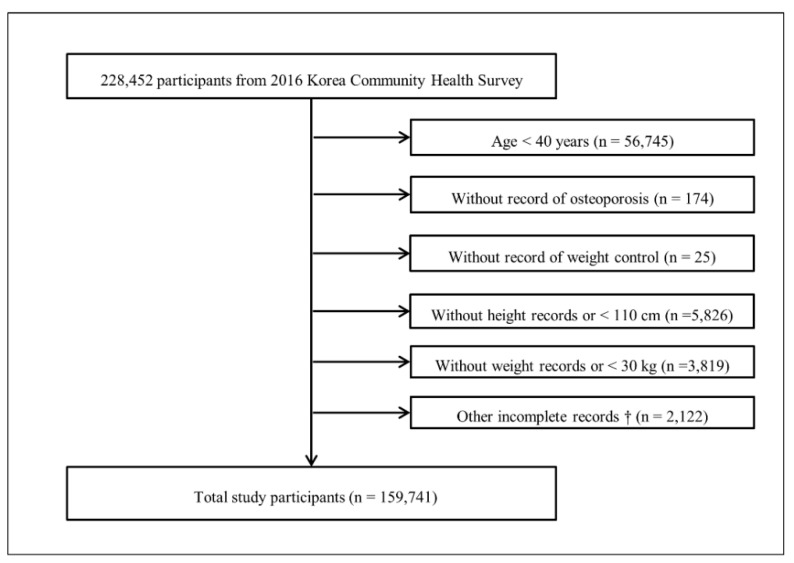
A schematic illustration of the participant selection process used in the present study. Of a total of 228,452 participants, 159,741 participants were selected after exclusion.

Of the 228,452 total participants, we excluded the following participants in this study: those who were <40 years old (n = 56,745); participants who did not report answers to osteoporosis questions (n = 174); participants who did not report answers to weight control (n = 25); participants who did not respond to height questions or whose height was <110 cm (n = 5826); participants who did not respond to weight questions or whose weight was <30 kg (n = 3819); and participants who did not complete records regarding income, education level, smoking, alcohol consumption, subjective health status, stress level, and physical activity (n = 2122). Ultimately, 159,741 participants were included in this study (Figure 1).

### 2.2. Definition of Osteoporosis

Participants were asked about their history of osteoporosis based on two questions. Osteoporosis was defined in two parts. In the first question, participants were asked if they were diagnosed with osteoporosis throughout their lifetime. In the second question, those who were diagnosed were also asked whether they were being treated currently. If the participants answered ‘yes’ to the first question, they were defined as ‘osteoporosis for entire life’, and others were considered as ‘non-osteoporosis for entire life’. If the participants answered ‘yes’ to the second question, they were defined as ‘current osteoporosis’ and others were considered as ‘non-current osteoporosis’.

### 2.3. Definition of Weight Control

Participants were asked ‘Have you tried to control your weight in the last year?’. The answer options were as follows: ‘Tried to lose weight’, ‘Tried to maintain weight’, ‘Tried to gain weight’, and ‘Never tried’. The answers were regrouped as three categories for simplification as follows: ‘Weight L&M’ (Tried to lose weight or Tried to maintain weight), ‘Weight gain’ (Tried to gain weight), and ‘Never tried’.

### 2.4. Covariate

Information on the region of residence, monthly income, education levels, smoking status, alcohol consumption, subjective health status, and physical activity intensity was gathered. The participants were asked “How often do you perform light or moderate leisure time physical activities for at least 10 min that cause only light sweating or a slight to moderate increase in breathing or heart rate?”. The participants reported on both the frequency and duration of moderate physical activities. To assess the amount of vigorous-intensity physical activity, subjective stress levels and obesity measured by BMI (body mass index, kg/m^2^) was surveyed as in our previous studies [13,14]. The patient’s height and weight were asked for directly, and BMI was calculated using this data. The formula for BMI is kg/m^2^, where kg is a person’s weight in kilograms and m^2^ is their height in meters squared.

### 2.5. Statistical Analysis

The general characteristics were compared according to weight control using Rao-Scott chi-square with sampling weights.

To calculate the odds ratios (ORs) with 95% confidence intervals (CIs) for osteoporosis for entire life and current osteoporosis, the crude model (simple model) and adjusted model (adjusted for age, sex, income, education level, region of residence, smoking, alcohol consumption, obesity, subjective health status, stress level, and physical activity) were analyzed using multiple logistic regression with sampling weights.

For subgroup analyses, we classified the participants according to sex (male; and female), age (40 to 59 years; and ≥ 60 years), and obesity (underweight; normal weight; overweight; and obese). ORs and 95% CIs of crude and adjusted models were calculated using multiple logistic regression with sampling weights. For the sample to be statistically representative of the population, the data collected from the survey were weighted by statisticians who performed post-stratification and considered the non-response rates [15].

Two-tailed analyses were conducted, and *p*-values below 0.05 were considered significant. The results were statistically analyzed using SAS version 9.4 (SAS Institute Inc., Cary, NC, USA). Sampling weights were used to conduct complex sampling design of the national survey using the survey procedure (PROC SURVEY).

## 3. Results

The analysis of general characteristics shows that older age, male, living in a rural area, smoker, lower-income level, non-alcohol consumer, lower education level, worse subjective health status, lower stress level, lower obesity, and lower physical activity are associated with the Never tried group (each *p* < 0.001). In addition, the higher prevalence of osteoporosis for entire life and current osteoporosis was associated with the Never tried group (each *p* < 0.001; Table 1).

In the Weight L&M group, the adjusted ORs for osteoporosis for entire life and current osteoporosis were 1.20 (95% CIs = 1.13–1.27, *p* < 0.001) and 1.16 (95% CIs 1.08–1.25, *p* < 0.001), respectively (each *p* < 0.001). The analyses stratified by obesity in osteoporosis for entire life were consistent in normal weight, overweight, and obesity groups, and results of analyses stratified by obesity in current osteoporosis were consistent in normal weight and overweight groups. In the Weight gain group, the adjusted ORs for osteoporosis for entire life and current osteoporosis were 1.83 (95% CIs = 1.64–2.05, *p* < 0.001) and 1.77 (95% CIs = 1.54–2.02, *p* < 0.001), respectively (each *p* < 0.001). The analyses stratified by obesity in both osteoporosis for entire life and current osteoporosis were consistent in underweight, normal weight, and obesity groups (Table 2).

In subgroup analyses according to age, sex, and obesity, the adjusted ORs for osteoporosis for entire life was higher in the Weight gain group than those in the Never tried group for all age and sex categories. In males ≥60 years of age, the adjusted ORs for osteoporosis for entire life were higher in the Weight L&M group than those in the Never tried group. The adjusted ORs for current osteoporosis were higher in the Weight gain group than those in the Never tried group for all age and sex categories, except for male 40 to 59 years of age (Table 3). The male to female ratio was higher for all age groups; among 40–59 years of age, it was 46.94% (39,685) men and 53.06% (44,851) women; and aged 60 years or older, it was 45.23% (34,014) men and 54.77% (41,191) women.

In females 40 to 59 years of age, the adjusted ORs for osteoporosis for entire life stratified by BMI were higher in the Weight gain group than in the Never tried group in the underweight, normal weight, and overweight groups. The adjusted ORs for osteoporosis for the entire life stratified by BMI were higher in the Weight L&M group compared to the Never tried group having normal weight. The adjusted ORs for current osteoporosis stratified by BMI were higher in the Weight L&M group compared to the Never tried group in those who were overweight (Table S1).

In male ≥60 years of age, the adjusted ORs for osteoporosis for entire life stratified by BMI were lower in the Weight L&M group compared to the Never tried group, for those who were underweight. The adjusted ORs for osteoporosis for the entire life and current osteoporosis stratified by BMI were higher in the Weight gain group than the Never tried group, for those of normal weight. The adjusted ORs for osteoporosis for entire life and current osteoporosis stratified by BMI were higher in both the maintenance group and the Weight gain group compared to the Never tried group, for those who were overweight. The adjusted ORs for osteoporosis for entire life stratified by BMI were higher in the Weight L&M group compared to the Never tried group, for those in the obesity group. The adjusted ORs for current osteoporosis stratified by BMI were higher in the Weight L&M group compared to the Never tried group, for those who were underweight (Table 3).

In females ≥60 years of age, the adjusted ORs for osteoporosis for entire life and current osteoporosis stratified by BMI were higher in the Weight gain group than the Never tried group in the underweight, normal weight, and obesity groups. The adjusted ORs for osteoporosis for entire life stratified by BMI were higher in the Weight L&M group compared to the Never tried group in the overweight and obesity groups. The adjusted ORs for osteoporosis for entire life stratified by BMI were higher in the maintenance group compared to the Never tried group, for those in the obesity group (Table 3).

## 4. Discussion

In our study, it was suggested that intention to change body weight may be associated with osteoporosis. In our study, the OR for osteoporosis in the Weight L&M group was higher than in the control group. Unexpectedly, the OR for osteoporosis in the Weight gain group was also higher than in the control group.

In our study, 47.6% of participants tried to lose and maintain weight in the previous year, slightly lower than the 64.7% of the meta-analysis [4]. The proportions of patients by weight indicate that 35.1% having a normal weight, 52.4% being overweight, and 66.9% being obese tried to lose or maintain weight in our study. Regarding incidence of osteoporosis, the incidence of osteoporosis for entire life was 7.8% (range, 6.5 to 10.8%) in our study. Similarly, previous authors estimated that 10.3% of adults 50 years and older in the USA have osteoporosis at the femoral neck or lumbar spine [16]. The findings mentioned above provide a basis for using the KCHS data to establish the weight change and incidence of osteoporosis. This result indicates that, in people of average weight, trying to change their weight can lead to osteoporosis. Meyer et al. assessed the effect of weight change on the risk of osteoporosis in 1476 middle-aged men [17]. The proportion of persons with osteoporosis was 6.2% for who lost or gained <5% of their body weight, and increased to 14.1% for those who lost 5 to 10%, and to 15.1% who lost over 10% of their body weight. The authors also obtained the same results after adjusting for several variables that may affect weight loss. These previous results are consistent with our study because these adjustments may attenuate the effect of intentional weight loss.

The OR for osteoporosis in the Weight L&M group was higher than in the control group in our study. Intentional weight loss may involve exercise, which have some bone-sparing effects, even among older adults [18]. However, a recent meta-analysis reported a significant effect of intentional weight loss on the decrease in hip BMD (MD −0.008, 95% CI −0.09 to −0.006 g/cm^2^, *p* < 0.001) and lumbar BMD (MD −0.018 g/cm^2^, 95% CI −0.019 to −0.017, *p* < 0.001) [7]. However, the subjects in the meta-analysis followed diet or exercise therapy according to a planned program, and the results may be different from real-world data due to an individual’s efforts. Moreover, because changes in BMD alone cannot determine whether osteoporosis occurs, these results cannot be easily compared with our study. A weight loss of 10% was shown to have a positive effect on the comorbidities associated with obesity, but to result in a 1% to 2% bone loss in the hip and total body, and a 3% to 4% loss at highly trabecular sites [19].

In our study, the OR for osteoporosis in the Weight gain group was higher than in the control group. There is lack of data on the relationship between weight gain attempts and osteoporosis, and we can gain insight from studies examining obesity and BMD. Obesity has previously been considered a protective factor for osteoporosis, but is not confirmed by large population-based studies [20]. The Study of Osteoporotic Fractures showed a U-shaped relationship between BMI and fracture risk after BMD adjustment, confirming that the effect of BMI on fracture risk is nonlinear [21]. Moreover, those who desire to gain weight may have other characteristics. Ganson et al. reported that those who underperceived their weight tended to try gain more weight [22]. Previous authors also found that 1 in 6 men attempting to gain weight had a normal range of BMI. These findings suggest that these individuals may increase the risk of disordered eating behaviours or unhealthy excess weight gain [22]. Addition insight is provided by a study that examined BMD in patients with bulimia nervosa, a disease of overeating. A meta-analysis found lower spine BMD in bulimia nervosa (standardized mean difference [SMD], −0.472; 95 % CI, −0.688, −0.255; *p* < 0.0001) [10]. More research is needed about whether diet adjustments to gain weight can affect osteoporosis.

When we stratified by obesity, the results were consistent in those having normal weight but not in other groups. This result means that, in people of average weight, trying to change their weight can lead to osteoporosis. When trying to lose weight in the underweight group, the OR of osteoporosis was not statistically significant. However, caution is required in interpreting this result because the number of underweight subjects among the total was very small (6289). An increase in osteoporosis appears to be a natural result in the underweight group when an effort is made to gain weight, because it has previously been shown that osteoporosis is common in underweight subjects [23]. The normal weight, overweight, and obese groups included subjects who desired to gain weight, and the OR of osteoporosis was significantly high in normal weight and obese people. These are likely people who underperceived their weight [22] or have dietary problems, and osteoporosis may have been caused by eating disorders [10]. Kammire et al. analyzed the results of intentional weight loss in 77 older adults having obesity. The authors found that loss of hip BMD persists in the year following a weight loss intervention [24]. Although only 1 in 6 adults having obesity reported maintaining weight loss [25], BMD is not recovered by regaining weight, and bone loss can continue to occur after weight loss ends. This suggests that even people who are overweight or obese should be careful about the effects of osteoporosis due to weight loss.

The results of subgroup analysis were not consistent when we stratified by age and sex. The ORs for osteoporosis were not significant among men aged 40–59 years and women who tried to lose and maintain their weight. The incidence of osteoporosis before the age of 60 is significantly lower than after 60 years old, and this appears to have been reflected in the current study [2]. Over the age of 60 years, the ORs were significantly increased only in males, in osteoporosis for entire life. In females, the ORs were significantly decreased before adjustment, but did not maintain their significance after adjustment. Data values can be distorted by several variables. By adjusting for these variables, we believed we obtained more accurate results. There are few data on the changes in BMD during weight loss in men and women [26,27]. In a study of overweight and obese participants, dietary intervention exhibited different results in men and women [27]. Men showed an increase in spine BMD, premenopausal women showed a decrease in femoral neck BMD, and postmenopausal women showed a decrease in spine and femoral neck BMD after intervention. However, the previous study was conducted only on overweight and obese subjects, so caution is needed in its interpretation.

There are several limitations in this study. First, we included the participants who tried to change their weight, but the method of weight change could not be identified in detail. Second, the number of subjects in the underweight group was very small, and it may be related to inadequate statistical power. Therefore, these results should be interpreted with caution. Third, according to the weight control attempts, there may have been errors because we relied fully on participant’s self-reported memory. There may be bias because we regrouped participants into three categories based only on simple questions. Moreover, osteoporosis patients were defined based on subjects’ answers to questions, rather than by diagnosis codes or diagnostic criteria. However, the KCDC has continually conducted KCHS surveys each year since 2008, and there is an advantage in their continuity. Moreover, due to the high level of education and low hospital thresholds in Korea, it is very easy to be diagnosed at a hospital, so many people are aware of osteoporosis. Fourth, although we adjusted for numerous covariates, several important confounding factors were not included. Because the present study used a questionnaire, the BMD and T-score could not be considered. Various variables may contribute to osteoporosis, such as the use of drugs that affect bone metabolism, type of hormone therapy or vitamin supplementation, and menopause and the time elapsed since menopause. Moreover, individual’s underlying diseases, history of fractures and history of surgery, and other individual disorders were not included. The incidence of osteoporosis may have been underestimated in men because osteoporosis is an under-recognized problem in men. However, we demonstrated that osteoporosis was significantly associated with weight control attempts regardless of the osteoporosis state. Moreover, our data demonstrated ORs in all subgroups stratified by groups according to age and weight control attempt. Further studies based on additional confounding factors and covariates using prospective study designs are required. Fifth, because of the cross-sectional design, the causal relationship between osteoporosis and weight control is unclear. However, one of the greatest strengths of this study is the large sample size of 159,741 participants, resulting in strong statistical power. Prospective studies and prospective follow-up studies with long follow-up times are essential. The sixth limitation is that the questions about exercise time in the questionnaire are not varied.

Despite the limitations of this study, the results are important for a variety of reasons. The biggest strength of our study is that the results were calculated by adjusting according to the degree of obesity. People who desire to lose weight will often be obese, and people who wish to gain weight will often be underweight. Most previous studies have failed to reflect this point and may have yielded distorted results. The present study demonstrated that the association between weight change attempts and osteoporosis may differ by age and sex group. Our study demonstrated the OR of weight change attempts and the history and current state of osteoporosis using a large sample. The used cohort was also a low-risk, asymptomatic large population of Koreans, which is an additional strength of our study. This research can be used as a reference for doctors to inform patients who attempt to change their weight about the adverse effects of such a change on their bone health.

## 5. Conclusions

In conclusion, our study suggested that intention to change body weight may be associated with osteoporosis after adjusting for confounding factors. In subgroup analyses according to weight, statistical significance was observed only in the normal weight group.

## Figures and Tables

**Table 1:** General characteristics of the participants.

Characteristics	Weight Control				*p*-Value
	Total	Weight L&M	Weight Gain	Never Tried	
Number (n, %)	159.741 (100.0)	76.061 (47.6)	8.431 (5.3)	75.249 (47.1)	
Age (n, %)					<0.001 *
40–59	84.536 (64.5)	50.075 (73.1)	3.999 (60.0)	30.462 (53.4)	
≥ 60	75.205 (35.5)	25.986 (26.9)	4.432 (40.0)	44.787 (46.6)	
Sex (n, %)					<0.001 *
Males	73.699 (49.2)	29.958 (42.7)	5.053 (65.3)	38.688 (55.9)	
Females	86.042 (50.8)	46.103 (57.3)	3.378 (34.7)	36.561 (44.1)	
Income (n, %)					<0.001 *
Lowest	37.900 (14.5)	11.793 (10.1)	2.393 (17.5)	23.714 (20.1)	
Low-middle	57.329 (32.5)	26.230 (30.1)	3.144 (36.0)	27.955 (35.4)	
Upper-middle	39.493 (30.4)	22.428 (33.0)	1.819 (27.7)	15.246 (27.3)	
Highest	25.019 (22.6)	15.610 (26.8)	1.075 (18.7)	8.334 (17.3)	
Region of residence (n, %)					<0.001 *
Urban	71.853 (68.4)	39.037 (70.9)	3.424 (64.8)	29.392 (65.4)	
Rural	87.888 (31.6)	37.024 (29.1)	5.007 (35.2)	45.857 (34.6)	
Smoking (n, %)					<0.001 *
None	95.947 (57.7)	50.275 (63.6)	3.985 (43.2)	41.687 (51.4)	
Past smoker	33.502 (21.0)	14.715 (20.0)	1.972 (23.8)	16.815 (22.1)	
Current smoker	30.292 (21.3)	11.071 (16.4)	2.474 (32.9)	16.747 (26.5)	
Alcohol (n, %)					<0.001 *
None	57.904 (29.7)	23.369 (26.3)	3.255 (32.0)	31.280 (34.2)	
≤ 1 time a month	38.118 (25.6)	21.320 (28.9)	1.729 (22.4)	15.069 (21.6)	
2–4 times a month	28.126 (20.8)	16.055 (23.3)	1.325 (19.1)	10.746 (17.6)	
≥ 2 times a week	35.593 (23.8)	15.317 (21.5)	2.122 (26.4)	18.154 (26.6)	
Education level (n, %)					<0.001 *
Low	70.728 (30.7)	24.567 (22.9)	3.975 (32.5)	42.186 (41.1)	
Middle	51.351 (36.1)	27.943 (37.8)	2.736 (38.9)	20.672 (33.4)	
High	37.662 (33.2)	23.551 (39.3)	1.720 (28.6)	12.391 (25.5)	
Obesity (n, %)					<0.001 *
Underweight	6.289 (3.2)	727 (0.9)	1.648 (18.1)	3.914 (4.5)	
Normal weight	66.329 (41.3)	23.255 (32.5)	6.015 (73.1)	37.059 (49.3)	
Overweight	42.684 (27.1)	22.353 (29.4)	646 (7.4)	19.685 (26.4)	
Obese	44.439 (28.3)	29.726 (36.1)	122 (1.4)	14.591 (19.8)	
Subjective health status (n, %)					<0.001 *
Good	50.239 (34.6)	26.832 (37.4)	2.197 (29.3)	21.210 (31.3)	
Normal	70.416 (46.5)	35.550 (47.8)	3.457 (44.6)	31.409 (45.0)	
Bad	39.086 (19.0)	13.679 (14.8)	2.777 (26.1)	22.630 (23.7)	
Stress (n, %)					<0.001 *
No	40.255 (21.1)	16.294 (18.8)	1.816 (18.6)	22.145 (24.5)	
Some	83.015 (54.5)	42.430 (57.2)	4.148 (51.7)	36.437 (51.1)	
Moderate	31.271 (21.0)	14.947 (20.8)	2.066 (25.2)	14.258 (20.7)	
Severe	5.200 (3.5)	2.390 (3.2)	401 (4.6)	2.409 (3.7)	
Physical activity (n, %)					<0.001 *
0 min/week	90.155 (55.3)	38.761 (49.3)	4.581 (54.3)	46.813 (63.6)	
1–149 min/week	12.951 (9.6)	7.368 (10.8)	0.737 (10.2)	4.846 (7.9)	
≥ 150 min/week	56.635 (35.1)	29.932 (39.9)	3.113 (35.5)	23.590 (28.5)	
Osteoporosis for entire life (n, %)					<0.001 *
No	142.812 (92.2)	69.790 (93.5)	7.186 (89.2)	65.836 (90.6)	
Yes	16.929 (7.8)	6.271 (6.5)	1.245 (10.8)	9.413 (9.4)	
Current osteoporosis (n, %)					<0.001 *
No	150.324 (95.9)	72.880 (96.9)	7.684 (93.9)	69.760 (94.9)	
Yes	9.417 (4.1)	3.181 (3.1)	0.747 (6.1)	5.489 (5.1)	

Abbreviation: Weight L&M, weight loss and maintenance; * The Rao-Scott chi-square test was analyzed with sampling weights, significance at *p* < 0.05.

**Table 2:** Crude and adjusted odd ratios (95% confidence interval) of weight control for osteoporosis.

Weight Control	ORs for Osteoporosis for Entire Life				ORs for Current Osteoporosis			
	Crude	*p*-Value	Adjusted †	*p*-Value	Crude	*p*-Value	Adjusted †	*p*-Value
Total participants (n = 159,741)								
Weight L&M	0.67 (0.64–0.70)	<0.001 *	1.20 (1.13–1.27)	<0.001 *	0.60 (0.56–0.64)	<0.001 *	1.16 (1.08–1.25)	<0.001 *
Weight gain	1.17 (1.07–1.28)	<0.001 *	1.83 (1.64–2.05)	<0.001 *	1.22 (1.08–1.36)	0.001 *	1.77 (1.54–2.02)	<0.001 *
Never tried	1		1		1		1	
Underweight (n = 6289)								
Weight L&M	0.49 (0.39–0.60)	<0.001 *	0.91 (0.71–1.16)	0.442	0.55 (0.42–0.72)	<0.001 *	1.10 (0.80–1.51)	0.558
Weight gain	1.11 (0.95–1.30)	0.187	1.92 (1.59–2.31)	<0.001 *	1.10 (0.91–1.34)	0.323	1.88 (1.49–2.36)	<0.001 *
Never tried	1		1		1		1	
Normal weight (n = 66,329)								
Weight L&M	0.65 (0.60–0.70)	<0.001 *	1.12 (1.02–1.22)	0.020 *	0.61 (0.55–0.67)	<0.001 *	1.18 (1.05–1.33)	0.007 *
Weight gain	0.95 (0.85–1.06)	0.379	1.69 (1.49–1.93)	<0.001 *	0.99 (0.86–1.14)	0.866	1.67 (1.43–1.96)	<0.001 *
Never tried	1		1		1		1	
Overweight (n = 42,684)								
Weight L&M	0.87 (0.80–0.95)	0.002 *	1.36 (1.21–1.53)	<0.001 *	0.77 (0.68–0.87)	<0.001 *	1.26 (1.10–1.45)	0.001 *
Weight gain	0.74 (0.50–1.11)	0.144	1.25 (0.78–1.99)	0.361	0.80 (0.46–1.38)	0.421	1.32 (0.73–2.41)	0.362
Never tried	1		1		1		1	
Obese (n = 44,439)								
Weight L&M	0.70 (0.64–0.77)	<0.001 *	1.23 (1.10–1.38)	<0.001 *	0.59 (0.52–0.67)	<0.001 *	1.08 (0.94–1.25)	0.292
Weight gain	1.17 (0.63–2.16)	0.616	2.76 (1.33–5.76)	0.007 *	1.17 (0.62–2.23)	0.626	2.49 (1.24–5.02)	0.011 *
Never tried	1		1		1		1	

* Logistic regression with sampling weights, significance at *p* < 0.05; † Adjusted for age, sex, income level, education level, region of residence, smoking, alcohol consumption, obesity, subjective health status, stress level, and physical activity.

**Table 3:** Subgroup analysis of crude and adjusted odd ratios (95% confidence interval) of weight control for osteoporosis according to age and sex.

Weight Control	ORs for Osteoporosis for Entire Life				ORs for Current Osteoporosis			
	Crude	*p*-Value	Adjusted †	*p*-Value	Crude	*p*-Value	Adjusted †	*p*-Value
Age 40–59 years old, males (n = 39,685)								
Weight L&M	0.86 (0.61–1.22)	0.395	0.86 (0.59–1.26)	0.445	0.90 (0.51–1.60)	0.720	0.96 (0.52–1.77)	0.900
Weight gain	2.03 (1.25–3.31)	0.004 *	2.00 (1.22–3.28)	0.006 *	1.32 (0.61–2.84)	0.480	1.20 (0.54–2.63)	0.658
Never tried	1		1		1		1	
Age 40–59 years old, females (n = 44,851)								
Weight L&M	0.91 (0.80–1.03)	0.135	1.09 (0.96–1.24)	0.189	0.86 (0.72–1.03)	0.096	1.06 (0.88–1.28)	0.531
Weight gain	2.28 (1.79–2.91)	<0.001 *	2.04 (1.58–2.64)	<0.001 *	2.08 (1.44–2.99)	<0.001 *	1.76 (1.20–2.57)	0.004 *
Never tried	1		1		1		1	
Age ≥ 60 years old, males (n = 34,014)								
Weight L&M	1.04 (0.86–1.25)	0.713	1.29 (1.07–1.56)	0.009 *	0.87 (0.68–1.11)	0.253	1.18 (0.91–1.53)	0.223
Weight gain	1.80 (1.36–2.37)	<0.001 *	1.81 (1.36–2.40)	<0.001 *	1.89 (1.34–2.67)	<0.001 *	1.97 (1.38–2.81)	<0.001 *
Never tried	1		1		1		1	
Age ≥ 60 years old, females (n = 41,191)								
Weight L&M	0.80 (0.76–0.85)	<0.001 *	1.01 (0.95–1.08)	0.676	0.75 (0.70–0.81)	<0.001 *	0.96 (0.89–1.04)	0.344
Weight gain	1.64 (1.44–1.86)	<0.001 *	1.67 (1.47–1.90)	<0.001 *	1.71 (1.48–1.97)	<0.001 *	1.74 (1.50–2.02)	<0.001 *
Never tried	1		1		1		1	

Abbreviation: N/A, not applicable; Weight L&M, weight loss and maintenance; * Logistic regression was analyzed with sampling weights, significance at *p* < 0.05; † Adjusted for income level, education level, region of residence, smoking, alcohol consumption, obesity, subjective health status, stress level, and physical activity.

## Data Availability

The data included in this study are available from KCHS, but restrictions apply to availability. These data were used under a license for the current study only and are not publicly available.

## Supplementary Materials

The following are available online at https://www.mdpi.com/article/10.3390/ijerph182413368/s1, Table S1: Subgroup analysis of crude and adjusted odd ratios (95% confidence interval) of weight control for osteoporosis according to age, sex, and obesity.
