# Supplementary material for: Weight Change Is Associated with Osteoporosis: A Cross Sectional Study Using the Korean Community Health Survey
Source: Int J Environ Res Public Health. 2021 Dec 19;18(24):13368. doi: 10.3390/ijerph182413368 (PMC8707057; doi:10.3390/ijerph182413368)
Supplement: Supplementary file 1 [file ijerph-18-13368-s001.zip › ijerph-1473399-supplementary.pdf]

Table S1 Subgroup analysis of crude and adjusted odd ratios (95% confidence interval) of weight control for osteoporosis according to age, sex, and obesity

Weight control	ORs for osteoporosis for entire life				ORs for current osteoporosis			
	Crude	P-value	Adjusted †	P-value	Crude	P-value	Adjusted †	P-value
Age 40-59 years old, males, underweight (n = 456)								
Weight L&M	11.81 (8.88-15.69)	<0.001*	20.34 (11.72-35.28)	<0.001*	N/A	N/A	N/A	N/A
Weight gain	2.60 (2.01-3.37)	<0.001*	7.98 (4.20-15.13)	<0.001*	N/A	N/A	N/A	N/A
Never tried	1		1		1		1	
Age 40-59 years old, males, normal weight (n= 12,849)								
Weight L&M	0.80 (0.44-1.45)	0.458	0.76 (0.41-1.40)	0.372	1.32 (0.56-3.14)	0.528	1.25 (0.51-3.05)	0.630
Weight gain	1.51 (0.89-2.59)	0.130	1.53 (0.88-2.67)	0.135	0.90 (0.40-2.04)	0.800	0.85 (0.37-1.96)	0.709
Never tried	1		1		1		1	
Age 40-59 years old, males, overweight (n= 11,639)								
Weight L&M	1.23 (0.63-2.41)	0.549	1.41 (0.69-2.85)	0.347	0.64 (0.19-2.19)	0.480	0.60 (0.24-1.47)	0.264
Weight gain	N/A	N/A	N/A	N/A	N/A	N/A	N/A	N/A
Never tried	1		1		1		1	
Age 40-59 years old, males, obese (n= 14,741)								

Weight L&M	1.11 (0.54-2.26)	0.785	1.03 (0.46-2.31)	0.944	1.64 (0.48-5.54)	0.429	2.07 (0.51-8.36)	0.309
Weight gain	N/A	N/A	N/A	N/A	N/A	N/A	N/A	N/A
Never tried	1		1		1		1	
Age 40-59 years old, females, underweight (n= 1,751)								
Weight L&M	0.80 (0.55-1.17)	0.245	1.01 (0.69-1.50)	0.946	0.32 (0.13-0.80)	0.015*	0.38 (0.14-1.04)	0.061
Weight gain	3.19 (2.29-4.46)	<0.001*	2.10 (1.50-2.94)	<0.001*	2.17 (1.51-3.10)	<0.001*	0.97 (0.65-1.44)	0.883
Never tried	1		1		1		1	
Age 40-59 years old, females, normal weight (n= 22,207)								
Weight L&M	0.85 (0.72-0.99)	0.038*	1.19 (1.00-1.40)	0.048*	0.84 (0.66-1.07)	0.155	1.28 (0.99-1.65)	0.055
Weight gain	2.11 (1.55-2.88)	<0.001*	1.57 (1.13-2.18)	0.007*	1.88 (1.16-3.03)	0.009*	1.29 (0.78-2.13)	0.321
Never tried	1		1		1		1	
Age 40-59 years old, females, overweight (n= 10,709)								
Weight L&M	0.98 (0.75-1.29)	0.909	1.33 (0.99-1.78)	0.056	1.24 (0.87-1.76)	0.244	1.75 (1.20-2.53)	0.003*
Weight gain	2.83 (1.54-5.19)	<0.001*	2.37 (1.59-3.54)	<0.001*	N/A	N/A	N/A	N/A
Never tried	1		1		1		1	
Age 40-59 years old, females, obese (n= 10,184)								
Weight L&M	0.98 (0.76-1.27)	0.874	1.26 (0.97-1.63)	0.088	0.76 (0.53-1.08)	0.124	0.98 (0.69-1.39)	0.896

Weight gain	1.12 (0.13-9.72)	0.922	1.83 (0.30-11.23)	0.515	2.44 (0.27-21.81)	0.425	3.59 (0.61-21.08)	0.157
Never tried	1		1		1		1	
Age ≥ 60 years old, males, underweight (n= 1,591)								
Weight L&M	0.78 (0.67-0.92)	0.003*	0.68 (0.57-0.80)	<0.001*	0.99 (0.82-1.19)	0.899	1.26 (1.02-1.54)	0.030*
Weight gain	0.96 (0.81-1.12)	0.572	0.92 (0.77-1.08)	0.301	0.84 (0.66-1.06)	0.142	0.93 (0.70-1.23)	0.602
Never tried	1		1		1		1	
Age ≥ 60 years old, males normal weight (n= 13,843)								
Weight L&M	1.14 (0.84-1.53)	0.407	1.29 (0.96-1.75)	0.097	0.77 (0.48-1.23)	0.272	0.96 (0.60-1.53)	0.855
Weight gain	1.44 (1.03-2.00)	0.033*	1.62 (1.16-2.28)	0.005*	1.62 (1.13-2.32)	0.008*	1.97 (1.37-2.84)	<0.001*
Never tried	1		1		1		1	
Age ≥ 60 years old, males overweight (n= 9,991)								
Weight L&M	1.60 (1.27-2.02)	<0.001*	2.10 (1.64-2.70)	<0.001*	1.29 (0.94-1.77)	0.114	1.93 (1.42-2.63)	<0.001*
Weight gain	1.60 (1.18-2.15)	0.002*	1.56 (1.14-2.14)	0.006*	2.97 (2.15-4.10)	<0.001*	2.98 (2.14-4.15)	<0.001*
Never tried	1		1		1		1	
Age ≥ 60 years old, males obese (n= 8,589)								
Weight L&M	1.23 (0.91-1.67)	0.177	1.47 (1.08-2.00)	0.014*	1.03 (0.69-1.55)	0.876	1.20 (0.79-1.82)	0.387
Weight gain	0.61 (0.08-4.53)	0.631	0.67 (0.09-5.10)	0.697	1.15 (0.16-8.59)	0.890	1.32 (0.17-10.45)	0.790

Never tried	1		1		1		1	
Age ≥ 60 years old, females, underweight (n= 2,491)								
Weight L&M	0.76 (0.60-0.96)	0.021*	0.80 (0.62-1.02)	0.075	1.05 (0.81-1.37)	0.717	1.10 (0.84-1.45)	0.479
Weight gain	1.65 (1.40-1.95)	<0.001*	1.48 (1.26-1.74)	<0.001*	1.74 (1.43-2.12)	<0.001*	1.48 (1.25-1.75)	<0.001*
Never tried	1		1		1		1	
Age ≥ 60 years old, females, normal weight (n= 17,430)								
Weight L&M	0.82 (0.75-0.89)	<0.001*	1.04 (0.94-1.14)	0.476	0.81 (0.73-0.91)	0.001*	1.09 (0.97-1.22)	0.173
Weight gain	1.44 (1.25-1.66)	<0.001*	1.44 (1.26-1.66)	<0.001*	1.49 (1.28-1.75)	<0.001*	1.50 (1.28-1.75)	<0.001*
Never tried	1		1		1		1	
Age ≥ 60 years old, females, overweight (n= 10,345)								
Weight L&M	0.90 (0.81-0.98)	0.022*	1.19 (1.07-1.32)	<0.001*	0.81 (0.72-0.91)	0.001*	1.08 (0.95-1.22)	0.233
Weight gain	1.18 (0.74-1.89)	0.484	1.14 (0.70-1.85)	0.595	1.21 (0.64-2.29)	0.563	1.15 (0.60-2.20)	0.679
Never tried	1		1		1		1	
Age ≥ 60 years old, females, obese (n= 10,925)								
Weight L&M	0.84 (0.77-0.93)	<0.001*	1.12 (1.01-1.24)	0.032*	0.74 (0.66-0.84)	<0.001*	0.99 (0.87-1.13)	0.894
Weight gain	3.54 (1.66-7.53)	0.001*	3.80 (1.71-8.44)	0.001*	2.30 (1.06-4.97)	0.035*	2.44 (1.13-5.28)	0.023*
Never tried	1		1		1		1	

Abbreviation: N/A, not applicable; Weight L&M, weight loss and maintenance

* Logistic regression was analyzed with sampling weights, Significance at $P < 0.05$

† Adjusted for Income level, education level, region of residence, smoking, alcohol consumption, subjective health status, stress level, and physical activity
